# Negative effect of zoledronic acid on tendon-to-bone healing: In vivo study of biomechanics and bone remodeling in a rat model

**DOI:** 10.1080/17453674.2018.1440189

**Published:** 2018-03-01

**Authors:** Geir Aasmund Hjorthaug, Endre Søreide, Lars Nordsletten, Jan Erik Madsen, Finn P Reinholt, Sanyalak Niratisairak, Sigbjørn Dimmen

**Affiliations:** 1 Division of Orthopedic Surgery, Oslo University Hospital (OUS), Norway; 2 Institute of Clinical Medicine, Faculty of Medicine, University of Oslo (UIO); 3 Experimental Orthopedic Research, Institute for Surgical Research, OUS; 4 Department of Orthopedic Surgery, Martina Hansen’s Hospital; 5 Department of Pathology, OUS; 6 Biomechanics Laboratory, Division of Orthopedic Surgery, OUS; g Department of Orthopedic Surgery, Lovisenberg Diaconal Hospital, Norway

## Abstract

**Background and purpose:**

Outcome after ligament reconstruction or tendon repair depends on secure tendon-to-bone healing. Increased osteoclastic activity resulting in local bone loss may contribute to delayed healing of the tendon–bone interface. The objective of this study was to evaluate the effect of the bisphosphonate zoledronic acid (ZA) on tendon-to-bone healing.

**Methods:**

Wistar rats (n = 92) had their right Achilles tendon cut proximally, pulled through a bone tunnel in the distal tibia and sutured anteriorly. After 1 week animals were randomized to receive a single dose of ZA (0.1 mg/kg IV) or control. Healing was evaluated at 3 and 6 weeks by mechanical testing, dual-energy X-ray absorptiometry and histology including immunohistochemical staining of osteoclasts.

**Results:**

ZA treatment resulted in 19% (95% CI 5–33%) lower pullout strength and 43% (95% CI 14–72%) lower stiffness of the tendon–bone interface, compared with control (2-way ANOVA; p = 0.009, p = 0.007). Administration of ZA did not affect bone mineral density (BMD) or bone mineral content (BMC). Histological analyses did not reveal differences in callus formation or osteoclasts between the study groups.

**Interpretation:**

ZA reduced pullout strength and stiffness of the tendon–bone interface. The study does not provide support for ZA as adjuvant treatment in tendon-to-bone healing.

Tendons and ligaments attach to bone through a transitional fibrocartilage tissue, the enthesis. This transitional tissue is complex in composition and organization and is not regenerated during healing of injuries or surgical repair. Tendon and ligament injuries often require surgical repair or reconstruction to regain function, and a favorable outcome depends on solid tendon-to-bone healing (Harryman et al. 1991, Gulotta and Rodeo 2007, Ekdahl et al. 2008). The osteointegration of the graft is the weak link in early tendon-to-bone tunnel healing. In a randomized controlled trial, transient decrease in local bone mineral density (BMD) was observed in the knee region of patients undergoing anterior cruciate ligament (ACL) reconstruction (Lui et al. 2012). Bone loss has also been observed in experimental tendon–bone repair studies and is probably due to increased ostoclastic activity (Galatz et al. 2005, Rodeo et al. 2007). Furthermore, a negative correlation between local bone loss and strength of the tendon–bone interface has been reported (Silva et al. 2002). Bone surfaces with high osteoclastic activity and peri-tunnel bone loss may therefore become a less suitable scaffold for healing of the tendon graft. Thus, prevention of early bone loss could enhance tendon-to-bone healing and improve clinical outcomes.

Bisphosphonates (BPs) are rapidly incorporated into the crystal structure of apatite bone matrix (Cole et al. 2016) and bind to the bone present at the time of administration (Amanat et al. 2007). The main effect of BPs is inhibition of osteoclasts, resulting in increased BMD (Russell et al. 2008). Several experimental studies have reported that BP treatment may enhance fracture healing (Li et al. 2001, Amanat et al. 2005) and implant fixation (Andersson et al. 2010). Only a limited number of studies have assessed the effect of BPs using tendon-to-bone models, but improved healing has been reported for alendronate (Thomopoulos et al. 2007, Lui et al. 2013). Zoledronic acid (ZA) is the most potent BP available (Russell et al. 2008) and, to our knowledge, the effect of ZA on tendon-to-bone tunnel healing has not been tested.

We hypothesized that ZA treatment would improve the mechanical properties at 3 and 6 weeks of tendon-to-bone tunnel healing in rats by reducing the local bone loss.

## Materials and methods

### Animals

Female Wistar rats (n = 92) (Taconic Europe, Lille Skensved, Denmark), skeletally mature, mean weight of 238 g (SD 9), were included in the study. The animals were acclimatized for 2 weeks before the surgical procedure, and were kept 2 per wire-topped plastic cage in an accredited animal facility with controlled temperature (21 °C ± 1), humidity (55% ± 10), ventilation and 12 hours light/dark cycles. They were allowed free access to water and standard laboratory rodent nutrition. Analgesics were given according to protocol (Hjorthaug et al. 2015). Anesthesia was induced and maintained by intraperitoneal (IP) injection of ZRF cocktail. The induction dose was 1.6 mL/kg; a volume of 0.4 mL. Antibiotics were not used.

### Surgical procedure

The skin on the medial side of the right Achilles tendon was incised with a 10 mm longitudinal incision. The Achilles tendon was released proximally from the calf muscle while the calcaneal insertion distally was left intact. A drill hole was made in the distal tibia in a dorsoventral direction, 3 mm proximal to the ankle joint. Predrilling was done with a 1.0 mm drill bit followed by drilling with a 1.5 mm drill bit at 3,200 rpm. A holding suture was added to the released tendon in a Kessler suture fashion using a 2-string monofilament nonabsorbable suture. The sutures were guided through the tunnel from posterior to anterior and the tendon gently passed through the tunnel and sutured to the anterior soft tissue with the ankle joint in a 90° flexed position. The skin incision was closed using resorbable sutures and sealed with spray dressing. No restriction on weight bearing was applied postoperatively.

### Groups

7 days postoperatively, single animals were allocated to either treatment (ZA group) or control group by computerized randomization. Under general anesthesia, induced by a low dose of ZRF cocktail (0.1 mL) IP and maintained by isoflurane (1.5–2.5%) inhalation anesthesia, the animals in the treatment group received a single ZA dose (0.1 mg/kg, total volume 0.5 mL) by IV infusion (Aclasta, Novartis, Frimley, UK) in the tail vein (Amanat et al. 2007). Control animals received an injection of saline in the same volume and route. The personnel giving the infusions were blinded to groups, as were observers before all further analyses. The animals were killed after 3 or 6 weeks.

### Mechanical assessment

Animals allocated to mechanical assessments were killed by pentobarbital overdose. The right hind leg was disarticulated through the knee, and the fibula, talus, foot, and soft tissue including the long tendons were removed. The anterior suture was removed. The tibia, calcaneus, and tendon graft were carefully preserved and the specimens were tested and analyzed as described (Hjorthaug et al. 2015) in a material testing machine (Model 858 Mini Bionix with MTS FlexTest digital controller, MTS Systems Corporation, Eden Prairie, Minnesota, USA) in a custom-made jig, designed to firmly hold the tibia and calcaneus without applying pressure to the tendon insertion site. No pre-tensioning was done. A 250N load cell was pulled upwards at a constant speed of 0.1 mm/s. The sampling frequency was 20 Hz over 120 s. All specimens were pulled to complete failure. Data were analyzed for pullout strength, stiffness, energy, and elongation.

### Histological evaluation

Tissues of 5 animals from each study group at each time point were fixed in vivo by vascular perfusion of 0.1 M phosphate-buffered 2% paraformaldehyde during deep anesthesia. The right leg containing the tendon-bone-interface was stripped of the skin only. The 3rd tail vertebra was debrided of all soft tissues. Specimens were immersed in the same fixative as above for 2 days, and decalcified in 7% EDTA in a 0.1 M phosphate buffer containing 0.5% paraformaldehyde. The specimens containing the tendon-bone-interface were embedded in paraffin and serial sectioned with 5 µm section thickness from anterior to posterior.

#### Light microscopy

**Figure 1.**
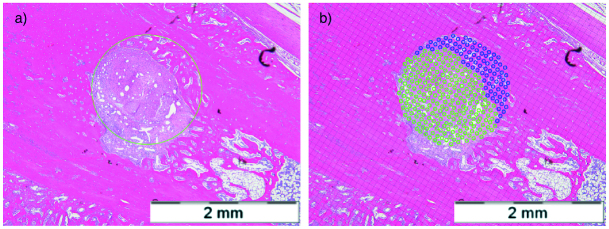
Medium power magnification (x10) light microscopy of a H&E stained histological section in a specimen from the 3 weeks ZA group from the middle of the bone tunnel showing (a) the superimposed circle of 1.6 mm and (b) the grid used to calculate the ratio of bone and tendon inside the tunnel. Scale bars =2 mm.

For semiquantitative bone morphometric analyses of the bone–tendon interface, 1 representative section centrally in the coronal plane was analyzed. Digital images were analyzed using AnalySIS V (Olympus Soft Imaging Solutions GmbH, Münster, Germany). A circle of 1.6 mm in diameter was superimposed to the tunnel profile, the chosen diameter being 0.1 mm larger than the used drill bit to compensate for any elliptical shaped appearance of the bone tunnel due to manual drilling and the manual sectioning along the axis of the tunnel. Mineralized tissue formation inside the bone tunnel was measured. The bone–tendon ratio inside the tunnel was calculated by point counting using a square test grid (Figure 1).

#### Histochemistry

**Figure 2.**
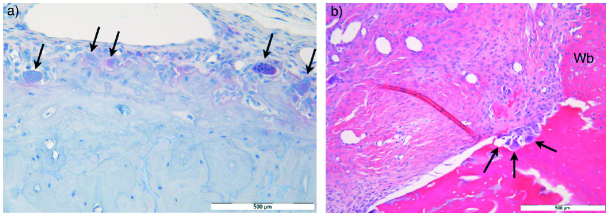
(a) High-power magnification (x40) light microscopy of section of the calcaneus from a specimen in the ZA group after 3 weeks subjected to TRAP enzyme histochemistry hematoxylin nuclear staining. Arrows indicate counted osteoclasts. (b) High-power magnification (x40) light microscopy of H&E stained section of the tendon–bone interface from a specimen in the control group after 6 weeks with bone resorption lacunae (arrows) in the tunnel wall containing osteoclasts. There is adjacent new woven bone (Wb) formation. Scale bars =500 µm.

To identify and estimate the number of osteoclast profiles in the paraffin sections, a standard protocol for the osteoclast marker tartrate-resistant acid phosphatase (TRAP) was used as suggested by the provider of a commercially available kit (Acid Phosphatase Leukocyte, TRAP, 387A-1KT lot SLBB7136, Sigma Aldrich). The regions of interest (ROI) were the bone tunnel, the ipsilateral calcaneus and the 3rd tail vertebra. The analyses were performed both on unstained and on sections stained with hematoxylin as nuclear stain. The TRAP staining was graded as strong, weak, or none, and osteoclast profiles were counted. Osteoclasts were defined as TRAP-positive cells containing more than 1 nucleus (Figure 2). Tissue from a previous study was used as positive control (Solberg et al. 2015).

### Bone mineral measurement and radiographic evaluation

**Figure 3.**
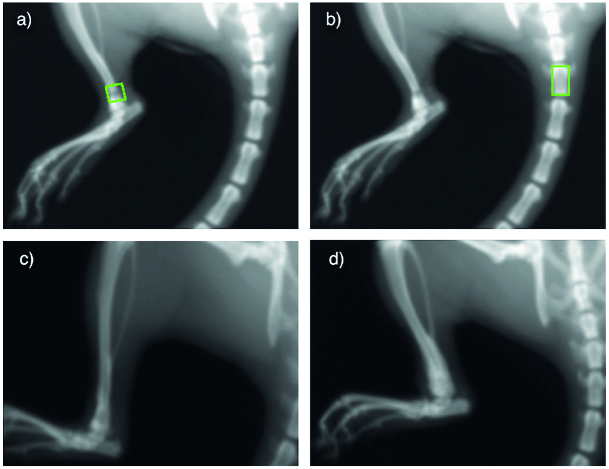
Upper panel: A specimen at baseline, showing the region of interest used for BMD and BMC measurements over the bone tunnel (a) and 3rd tail vertebra (b). Lower panel: X-rays of two 6-weeks specimens demonstrating bone formation around the bone tunnel in a specimen from the control group (c) and the ZA group (d). Radiographs were obtained from Lunar PIXImus.

Under anesthesia at each time point in all animals, BMD and bone mineral content (BMC) were measured over the bone tunnel using DEXA (Lunar PIXImus, software v. 2.10, Lunar, Madison, WI, USA). The ROI consisted of 19 x 19 pixels (≈ 4 x 4 mm) that was aligned along the axis of the bone tunnel and placed flush with the anterior bone cortex. To assess systemic bone mineral effects, we used a secondary ROI, 19 x 33 pixels (≈ 4 x 7 mm), that was aligned to the 3rd tail vertebra (Figure 3). Radiographs were reviewed to reveal any complications.

### Statistics

The number of animals was based on previous studies, and estimated based on our main outcome (Dimmen et al. 2009, Hjorthaug et al. 2015). An a priori power calculation for the sample size was not performed. The main outcome was pullout strength. Other outcomes were stiffness, energy absorption, elongation, and differences in BMD and BMC from baseline. 2 independent observers analyzed these 6 variables, and the mean values were calculated and used for the statistical analyses. Normality of the distribution was evaluated using histograms with normality curves. Variances were tested by Levenes’ test, and in case of detected heterogeneity, comparisons for group mean differences with 95% confidence intervals (CI) were calculated on log-transformed data of all above outcomes except elongation. Differences between the groups were tested by 2-way Analysis of Variance (ANOVA), with time and treatment as fixed factors. If no interaction effects were found, analysis of the main effects for time and treatment was performed. Pairwise comparisons were run where effect estimates were expressed as a percentage of the mean of the corresponding group and the 95% confidence intervals (CI) and p-values were Bonferroni-adjusted to correct for multiple comparisons. The alpha level was set to 0.05. Results for semiquantitative data from histology are given as medians and ranges, and the groups were compared using the non-parametric Mann–Whitney test for independent samples, without correction of the p-values. Statistical analyses were performed using IBM SPSS® Statistics for Macintosh v. 23.0 (IBM Inc., Chicago, Illinois, USA).

### Ethics, funding, and potential conflicts of interest

The experimental protocol was reviewed and approved by the Norwegian Animal Research Authority (2014, ID 5839), and the study conducted according to guidelines provided by Norecopa. The study was funded by internal hospital research grants. One author declares the following potential conflict of interest or source of funding: GAH received a minor research grant from Smith & Nephew in 2012.

## Results

### Animal inclusion

The body weight of all animals increased during the study period by mean 13.9 g (SD 6.4) and 25.3 g (SD 8.5), at 3 weeks and at 6 weeks respectively. 7 animals died perioperatively and 2 animals were killed due to wound complications during the first week. 83 animals were randomized to study groups. 2 animals died in the second anesthesia prior to injections 1 week postoperatively. Thus, 81 animals were available for final analysis including DEXA measurements. Of the 81 animals, 20 were selected for histology and 61 for mechanical tests. No fractures were observed clinically or on radiographs.

### Mechanical assessment

The tendons of all specimens were completely pulled out, and they all ruptured at the tendon–bone tunnel interface. No fractures occurred during testing. Overall, mean pullout strength was 5.7 N (SD 2.7) at 3 weeks and 9.4 N (SD 5.2) at 6 weeks. Interaction effects of “time” or “treatment” were not found in any of the mechanical variables (2-way ANOVA), therefore main effects were calculated. Pullout strength increased from 3 to 6 weeks by 22% (CI 9–40%) (p = 0.002) in ZA-treated animals and controls. Stiffness and energy increased similarly by 40% (CI 12–70%) (p = 0.007) and 18% (CI 4–35%) (p = 0.02), respectively. Elongation did not increase with time (p = 0.7).

**Figure 4.**
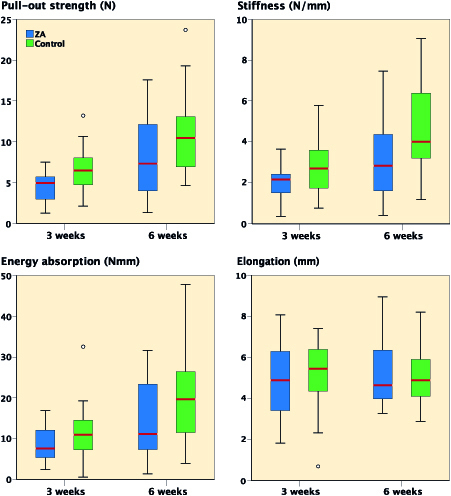
Clustered boxplots of data from mechanical testing of the tendon–bone interface. Except for elongation, all variables increased with time. There was a negative effect of ZA treatment in pullout strength and stiffness, but no significant differences in energy or elongation.

The main effect of ZA treatment was a reduction of pullout strength compared with control by 19% (CI 5–33%) (p = 0.009) at 3 and 6 weeks. ZA treatment reduced stiffness by 43% (CI 14–72%) (p = 0.004). No statistically significant differences for treatment between the groups were observed in energy (p = 0.1) or elongation (p = 0.9) (Figure 4).

### Bone mineral measurements

**Figure 5.**
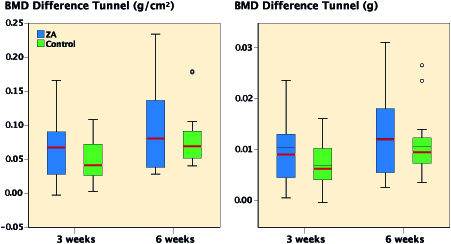
Clustered boxplots of data from DEXA measurements of the ROI over the bone tunnel: BMD and BMC difference from baseline.

Baseline BMD over the bone tunnels of the 81 included animals was mean 179 mg/cm2 (SD 24). Interaction effects between time and treatment were not found in any of the bone mineral variables, therefore main effects were calculated. BMD difference from baseline increased from 3 to 6 weeks by 15% (CI 3–27%) (p = 0.01). However, administration of ZA did not affect BMD (9%) (CI –4% to 21%) (p = 0.2) or BMC (p = 0.9) (Figure 5). No systemic differences in bone mineral, as measured over the 3rd tail vertebra for time or treatment, were detected.

### Histological findings

**Figure 6.**
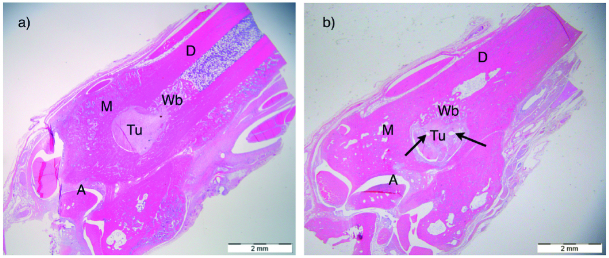
Low-power magnification (x4) light microscopy of H&E stained sections of distal leg showing anatomy and changes over time. The location of the bone tunnel (Tu) containing tendon graft was aimed 3 mm above the ankle joint in the transition between the tibia diaphysis (D) and metaphysis (M). The ankle joint (A) was visible in most sections. (a) 3-weeks ZA group: sharp tunnel edges. Callus/Wb. (b) 6-weeks control-group: Callus/Wb mature. The arrows point at large capillaries inside the tendon graft. Scale bars =2 mm.

**Figure 7.**
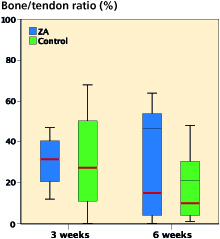
Clustered box-plots showing the bone–tendon ratio in the tunnel as measured by histomorphometry. No statistically significant effect of ZA treatment was observed at 3 weeks (p = 0.9, n = 8) or 6 weeks (p = 0.7, n = 9). Comparison of median values by independent samples Mann–Whitney test.

1 specimen (6 weeks control group) was excluded due to technical problems with decalcification, leaving 19 specimens for histological evaluation. All bone tunnels were located in the metaphysis, no tunnels extended into the ankle joint, no fractures were observed and heat necrosis was not pronounced. A moderate amount of periosteal callus was observed around the tibia at both 3 and 6 weeks. New woven bone formation appeared also in the diaphyseal bone marrow canal and inside the tunnel, sometimes replacing the tendon graft. The 6 weeks specimens demonstrated increased vascularity of the tendon graft in addition to more mature new bone formation (Figure 6). The inner surfaces of the bone tunnels were more irregular in the 6 weeks specimens. No evident tunnel widening or peri-tunnel bone loss was seen. Bone resorption lacunae were seen in both groups (see Figure 2). However, the ZA specimens were not histologically distinguishable from the control group in the H&E sections. The bone morphometric evaluation of new bone formation in the bone tunnels demonstrated no statistically significant differences between the groups in bone–tendon ratio at 3 or 6 weeks (Figure 7).

At 6 weeks, we noted both weak and strong TRAP-stained osteoclasts in the sections containing the bone tunnels. However, we could not detect differences between the ZA group and the controls, either regarding the number (Table) or the histologic distribution of osteoclasts. In the sections of 3rd tail vertebra, TRAP-positive osteoclasts were hardly present in any group.

**Table:** Total number of TRAP-positive osteoclast counts (median, range) in distal tibia containing the bone tunnel and the calcaneus of the ipsilateral leg. Comparison of median values by independent samples Mann–Whitney test. 1 outlier (^a^) of the 3-week controls was excluded from the above analyses due to a very high number of osteoclasts (> 200) in the calcaneus

	Distal tibia	Calcaneus
3 weeks		
Control**^a^** (n = 4)	Not measured	2 (1–4)
Zoledronic acid (n = 5)	Not measured	5 (4–15)
p-value		0.1
6 weeks		
Control (n = 4)	6 (5–23)	4 (1–4)
Zoledronic acid (n = 5)	17 (0–45)	6 (0–23)
p-value	1.0	1.0

## Discussion

Our study demonstrated an unfavorable effect of a single dose of ZA on pullout strength and stiffness of the tendon–bone interface. We were not able to detect any differences in bone remodeling by DEXA, or histology with specific osteoclast staining and bone morphometric evaluation of mineralized tissue inside the bone tunnels, but the sample size of specimens evaluated by histology was small.

The early phase of tendon to bone healing is associated with regional bone loss and decreased mechanical strength, especially if a bone-tunnel technique is used, as described in a canine model of toe flexor injury and repair (Silva et al. 2006). In a subsequent study, the same group was able to prevent the regional bone loss and improve tendon-to-bone repair strength at 3 weeks by administration of the BP alendronate (Thomopoulos et al. 2007). In a study in rats, alendronate was reported to reduce peri-tunnel bone loss as measured by CT (Lui et al. 2013). This prevention of local bone loss correlated with increased mechanical strength, but no differences in stiffness were observed. We used an unrestricted model and preserved the fibrous and callus tissue surrounding the tendon to increase external validity. The suture was removed prior to mechanical testing, to ensure that we assessed the healing and not the fixation and/or healing. We did not confirm the positive BP effects reported in these two studies (Thomopoulos et al. 2007, Lui et al. 2013).

ZA treatment increased the native supraspinatus tendon failure stress and improved bone density at the rotator cuff footprint in ovariectomized rats after 12 weeks (Cadet et al. 2010), but may not be directly comparable to the tendon-to-bone tunnel healing in our study.

We chose the ZA dose, route, and delayed administration based on a study of fracture healing in rats (Amanat et al. 2007). They argued that delayed administration of the anti-catabolic ZA would allow bone formation to establish, and increase the net difference between anabolism and catabolism by an increased uptake of ZA in the forming callus. Delayed administration of ZA has been used also in clinical bone-healing studies for the same reasons (Harding et al. 2010, 2011). However, the optimal dose and timing of ZA treatment in the presence of a fracture is not firmly established, and for tendon–bone healing it has not been much investigated.

There are some limitations to our model. The distal location for the bone tunnel on the tibia may provide limited healing potential with a reduced availability of marrow-derived mesenchymal stem cells due to the higher fat content of the marrow in the ankle region compared with the knee region. Nevertheless, the model may be relevant also for tendon transfers in foot/ankle surgery.

No clinical studies of BPs on tendon-to-bone healing exist. BMD was found to be one of the independent factors predicting tendon-to-bone healing in rotator cuff surgery (Chung et al. 2011), and local bone loss in the humeral head probably leads to a further decrease in cuff tendon insertion strength (Chen et al. 2015). Thus, the rationale for using bone resorption inhibitors as adjuvant treatment in tendon-to-bone healing remains plausible.

BPs in studies of fracture healing produce a larger callus and improved early mechanical fracture properties (Turker et al. 2016). In the early fracture-healing phase, a larger callus may explain an increased ability to withstand load. In the tendon-to-bone tunnel interface, the anatomical space for callus volume formation is limited. We were not able to observe delayed bone remodeling or prevention of local bone loss in the ZA-treated animals. If osteoclasts were inhibited by ZA a possible effect could be delayed transition from woven bone to lamellar bone, resulting in reduced tendon-to-bone healing.

In summary, our study of early tendon-to-bone tunnel healing in rats demonstrated that a single dose of ZA reduced pullout strength and stiffness of the tendon–bone interface. Local bone loss was not evident, and no difference in BMD between ZA-treated animals and controls at 3 or 6 weeks was noted. Our study does not provide evidence supporting the use of ZA as adjuvant treatment in tendon-to-bone healing.

The authors would like to thank the Department of Comparative Medicine, Oslo University Hospital, Rikshospitalet for providing excellent animal facilities and enthusiastic personnel at our disposal. Senior Engineer Linda T. Dorg is acknowledged for excellent help with the histological work.

GAH, ES, LN, JEM, FPR, and SD were responsible for the design of the study. GAH performed surgery, DEXA measurements, mechanical testing, histological analyses, independent DEXA analyses, statistical calculations, and wrote the draft manuscript. ES performed surgery, DEXA measurements, and independent DEXA analyses. JEM and SD performed surgery and DEXA measurements. FPR supervised histological analyses. SN performed independent mechanical analyses. All authors approved the final version of the manuscript.
